# FastPtx: a versatile toolbox for rapid, joint design of pTx RF and gradient pulses using Pytorch’s autodifferentiation

**DOI:** 10.1007/s10334-023-01134-7

**Published:** 2023-12-08

**Authors:** Dario Bosch, Klaus Scheffler

**Affiliations:** 1 High-Field MR Center, Max Planck Institute for Biological Cybernetics, 72076 Tübingen, Germany; 2 Department for Biomedical Magnetic Resonance, University of Tübingen, 72074 Tübingen, Germany

**Keywords:** Magnetic resonance imaging, Algorithms, MRI pulse design, Parallel transmission

## Abstract

**Objective:**

With modern optimization methods, free optimization of parallel transmit pulses together with their gradient waveforms can be performed on-line within a short time. A toolbox which uses PyTorch’s autodifferentiation for simultaneous optimization of RF and gradient waveforms is presented and its performance is evaluated.

**Methods:**

MR measurements were performed on a 9.4T MRI scanner using a 3D saturated single-shot turboFlash sequence for $B_1^+$ mapping. RF pulse simulation and optimization were done using a Python toolbox and a dedicated server. An RF- and Gradient pulse design toolbox was developed, including a cost function to balance different metrics and respect hardware and regulatory limits. Pulse performance was evaluated in GRE and MPRAGE imaging. Pulses for non-selective and for slab-selective excitation were designed.

**Results:**

Universal pulses for non-selective excitation reduced the flip angle error to an NRMSE of (12.3±1.7)% relative to the targeted flip angle in simulations, compared to (42.0±1.4)% in CP mode. The tailored pulses performed best, resulting in a narrow flip angle distribution with NRMSE of (8.2±1.0)%. The tailored pulses could be created in only 66 s, making it feasible to design them during an experiment. A 90° pulse was designed as preparation pulse for a satTFL sequence and achieved a NRMSE of 7.1%. We showed that both MPRAGE and GRE imaging benefited from the pTx pulses created with our toolbox.

**Conclusion:**

The pTx pulse design toolbox can freely optimize gradient and pTx RF waveforms in a short time. This allows for tailoring high-quality pulses in just over a minute.

## Introduction

Modern ultra-high field (UHF) MRI scanners are often equipped with parallel transmit (pTx) systems to overcome the inherent inhomogeneities of the transmit (Tx) radio-frequency (RF) field. These systems can drive multiple RF Tx elements independently. State-of-the-art pTx applications optimize individual RF waveforms for each RF channel, while also applying a gradient waveform at the same time [1–3]. In many cases, this gradient waveform is either predefined or generated through an earlier optimization step, in which a parameterized waveform was optimized [4, 5]. This reduces computational complexity, thus resulting in reduced calculation times. In recent works, gradient waveforms have been optimized using methods such as spline optimization or free optimization, together with a single-channel RF waveform [6, 7].

Universal pulses (UPs) are another way to reduce the time required for pTx pulse calculation. UPs are optimized using a database of transmit RF field ($B_1^+$) maps from a representative subject cohort, again usually on top of a predefined or parametrized gradient waveform. They allow for a pulse that performs well across the entire database. This eliminates the need for $B_1^+$ mapping and pulse calculation for individual subjects. However, UPs may not work as well for subjects who are not part of the representative cohort, and they are outperformed by subject-specific tailored pulses (TPs) in many cases. Despite these limitations, UPs have been shown to be effective in both non-selective and local excitation, as well as for inversion, saturation and refocussing [8, 9].

With modern optimization methods and the ever-increasing computational power even in mid-tier desktop computers, free optimization of parallel transmit pulses together with their gradient waveforms can be performed within reasonable time. Many optimization algorithms benefit from the availability of Jacobians of the target function. Providing explicit Jacobians for pulse optimization has proven to allow for more efficient optimization in the past [10]. This is, however, not possible in all cases, for example when exact Jacobians are not known or the exact optimization target changes too frequently, which would require additional code modifications. As an alternative, automatic differentiation can be employed. In this, derivatives of all operations on a variable are tracked during function evaluation and can be used to calculate the gradients of function inputs from the gradients of output arguments [11]. This allows users to define any function for forward-calculation without having to concern themselves with about the backward step, thus giving programmers additional flexibility.

In this work, we present a toolbox which uses the python framework PyTorch [12], originally designed for machine learning applications, for simultaneous optimization of RF and gradient waveforms. PyTorch’s autodifferentiation capabilities enable the automatic creation of Jacobian matrices for arbitrary functions, making optimization algorithms more efficient and reducing the time required for optimization. The PyTorch implementation of autodifferentiation allows for in-place operations, which makes it especially easy to use, and is highly efficient since its core implementation was done in C++. We show MPRAGE and GRE images acquired using a UP and a subject-specific TP, which was optimized in approximately 1 min using our toolbox.

### Theory

For optimizing pTx pulses, a forward simulation needs to be implemented. One option for this are Bloch simulations. While they require high computational power, they deliver accurate results for arbitrary RF and gradient waveforms. In a Bloch simulation, the values of individual magnetization vectors $\vec {m}$, also referred to as “Bloch spins”, are calculated. Beginning from an initial configuration such as equilibrium, the simulation is divided into discrete time steps. For each time step, a resulting magnetization vector is calculated by taking into account the effects of gradients, static magnetic field ($B_0$) offset, and RF. Additionally, the effects of $T_1$ and $T_2$ relaxation are computed.

1 $$\begin{aligned} \frac{d}{dt}\left( \begin{array}{c} M_x\\ M_y\\ M_z \end{array} \right) = \left( \begin{array}{ccc} -\frac{1}{T_2}&{} \gamma B_z &{} -\gamma B_y \\ -\gamma B_z &{} -\frac{1}{T_2}&{} \gamma B_x \\ \gamma B_y &{} -\gamma B_x &{} -\frac{1}{T_1} \end{array} \right) \left( \begin{array}{c} M_x\\ M_y\\ M_z \end{array} \right) + \left( \begin{array}{c} 0\\ 0\\ \frac{M_0}{T_1} \end{array} \right) \end{aligned}$$

Equation 1 shows the Bloch equations that describe the evolution of the magnetization vector for each time step in a rotating frame of reference. $M_x$, $M_y$, and $M_z$ represent the magnetization vector $\vec {m}$, $\gamma$ the gyromagnetic ratio, $T_1$ and $T_2$ the longitudinal and transversal relaxation times, $B_x$ and $B_y$ the real and imaginary component of the RF field in the position of the vector, which is a sum of the individual RF waveforms weighted by the complex $B_1^+$ sensitivities for the given position. $B_z$ represents the sum of all (spatially dependent) effects of the gradient fields and any $B_0$ inhomogeneities. $M_0$ is the equilibrium magnetization of $M_z$ and is often given in normalized units as 1. When implementing the Bloch simulation in software, the matrix multiplication can optionally be substituted by individual calculations for the individual components of $\vec {m}$. While being more laborious to implement, this allows for omitting individual steps if they are known to have little to no effect to reduce computation time.

**Table 1:** All terms that went into the cost function for optimizing RF and gradient pulses

Equation		Description
$pen_\text {rmse}(p,g)$	$= \sqrt{\displaystyle \overline{(|\alpha | - |\alpha _\text {target}|)^2}}$	Root mean square error of excited flip angle compared to target FA over all voxels
$err_\text {volt}(p)$	$= \max (0,|p|-U_\text {limit})^2 \cdot 10^6$	Positive non-zero value when maximum allowed pulse power is exceeded
$err_\text {maxG}(g)$	$= \max (0,|g|-G_\text {limit})^2 \cdot 10^{15}$	Positive non-zero value when maximum allowed gradient magnitude is exceeded
$err_\text {slew}(g)$	$= \max (0,|{\dot{g}}|-s_\text {limit})^2 \cdot 10^6$	Positive non-zero value when maximum allowed gradient slew rate (in [T/m/s] )is exceeded
$err_\text {edge}(p,g)$	$= \displaystyle \sum _{x,y,z}{\left( (g_{t=0} + g_{t=last}) \cdot 10^3\right) }^2$	Term to disallow the first or last gradient sample to be non-zero, which is a limitation of the MR scanner. By including this in the optimization, slew rate limits will still be observed by the optimizer
$pen_\text {power}$	$= \overline{(|p| \cdot 10^{-2})^2}$	Penalty for average pulse power
$pen_\text {slew}(g)$	$= \overline{|{\dot{g}}|^2} \cdot 10^{-6}$	Penalty for gradient slew rate
$err_\text {sar}(p)$	$= \max (0,sar-sar_\text {limit})^2 \cdot 10^2$	Positive non-zero value when maximum local SAR limit is exceeded

All terms depend on the gradient waveforms *g* [T/m], the RF pulse waveforms *p* [V], or both. $s_\text {limit}$ stands for the slew rate limit [T/m/s], $G_\text {limit}$ for the gradient magnitude limit [T/m], $U_{limit}$ for the voltage limit [V], and an overline ${\overline{x}}$ representing the arithmetic mean over all values of *x*

An alternative to Bloch simulations is following the so-called small tip angle approximation (STA). This assumes the angle by which $\vec {m}$ is flipped in each individual time step as well as in the entire pulse is small enough that $M_z$ can be approximated with its equilibrium value $M_0$. As a consequence, the resulting transverse magnetization becomes linearly dependent on the RF field: [13]

2 $$\begin{aligned} \frac{d}{dt}M_{xy} = -i \gamma B_z M_{xy} + i \gamma B_{xy} M_0 \end{aligned}$$

with

$$\begin{aligned} M_{xy}&= M_x + i M_y \\ B_{xy}&= B_x + i B_y \end{aligned}$$

Based on the STA, the *spatial domain method* accumulates the effects of the gradient waveforms (in the form of k-space trajectories), $B_0$ field offsets, and $B_1^+$ sensitivity maps into a system matrix *A* [1]. By reordering all complex RF waveforms into a single column vector $\vec {b}$, this allows for calculation of the transversal magnetization in a simple vector–matrix-multiplication:

3 $$\begin{aligned} \vec {m} = A \cdot \vec {b} \end{aligned}$$

With constant gradient waveforms, the system matrix *A* stays fixed and $\vec {b}$ can be optimized in a minimization problem with low computational requirements. When the gradient waveforms also need to be optimized, *A* has to be assembled during each step of the optimization process. While this increases computation time, the computational burden is still significantly lower than in Bloch simulations. This method, however, does not take $T_1$ and $T_2$ relaxation into account, nor will it be accurate for cases in which $M_z$ is reduced significantly.

## Methods

MR measurements were performed on a Magnetom 9.4T Plus MRI scanner (Siemens Healthineers, Erlangen, Germany) running Syngo VE12U software, equipped with a 16-channel pTx system (1kW RF power per channel) and a SC72 whole-body gradient system. A custom-built head array coil (16 transmit, 31 receive elements) was used [14]. The system was operated assuming the following hardware limitations: maximum RF magnitude: 185 V per channel; maximum gradient magnitude: 65 mT/m; maximum gradient slew rate: 185 T/m/s. Data was acquired from 14 healthy adults (age: 24...54 years, 9 male, 5 female). All experiments were performed with approval of the local ethics committee. Before each MR-experiment, informed signed consent was obtained from each volunteer.

### 1. Toolbox design and usage

For using the FastPtx toolbox, multiple steps are necessary as of now. All those steps are outlined here and will be explained in more detail in the following chapters.

Since pulse calculation relies on $B_1^+$ and $B_0$ maps, those need to be acquired. After reconstruction, they have to be stored in a MATLAB file, together with a binary mask of the region of interest, in our case a brain mask. $B_1^+$ fields have to be provided in [nT/V] and $B_0$ maps in [$\Delta$Hz]. The FastPtx toolbox is then executed by running a short python script, in which system limits, desired FA distribution and the desired number of iterations for optimization, as well as one or multiple files with $B_1^+$/$B_0$ maps are specified. The resulting optimized RF and gradient waveforms will be returned as PyTorch arrays by the FastPtx toolbox. They can either be used in custom code or passed onto another function of the FastPtx toolbox, which will write the pulse into a *.ini* file as required by the Siemens VE12U system we used. This file can then be transferred to the MRI scanner, for example via the local network.

The toolbox consists of three main modules. One module is called *io.py* and handles data in- and output. It includes functions for reading $B_1^+$ maps,$B_0$ maps, and VOP files, as well as functions to read and write pTx pulses in the *.ini* format required by the scanner. The second module, *smallTipAngle.py*, consists of a class that has methods for forward simulation, the cost function, and the optimization code. The third module, *bloch.py*, contains a class which is derived from smallTipAngle.py and overwrites the forward simulation methods. By doing so, most steps only needed to be implemented once even though both STA and Bloch simulation are supported.

### 2. Generation of $B_1^+$ maps

Individual-channel $B_1^+$ maps and a $B_0$ map of each subject were recorded with a centric-reordered 3D saturated single-shot turboFlash (3DsatTFL) sequence [15, 16]. A sinc pulse with a duration of 5 ms and a bandwidth of 4000Hz was used for saturation. RF interferometry [17] was used by phase-shifting one Tx channel by 180° at a time. Recovery time $T_{rec}$ between scans was 0.5 s for the relative $B_1^+$ scans and 7.5 s for all other scans. Other sequence parameters wereInterface: TR=2.44 ms, TE=0.75 ms, BW=700 Hz/Px, asymmetric echo, elliptical k-space filling, GRAPPA [18] 2x2, nominal flip angle(FA) excitation=2°, nominal FA presaturation=70°, $\tau$-excitation=200 µs, $\tau$-saturation=5000 µs, 795 k-space encoding steps, readout duration 1.94 s, matrix: 64$\times$64$\times$64, resolution 3.5 mm isotropic ($\tau$ indicates the pulse duration). Acquisition time: 197 s. An additional repetition with a longer TE was included in the sequence, which allowed for the estimation of $B_0$ maps. Based on the 3DsatTFL data, brain extraction was performed using a neural network [16].

### 3. RF pulse simulation

Software for pTx pulse optimization was developed in Python 3.9 using the PyTorch toolkit version 1.13.0. Local SAR supervision was performed using a Virtual Observation Points (VOP) model [19] with 208 VOPs, which was specifically generated for the RF coil in use. For VOP generation two voxel models (Duke and Ella, virtual family [20]) were simulated using CST Microwave Studio. VOP compression was performed using the compression algorithm by Orzada et al. [21]. The worst-case overestimation factor of the resulting VOPs was 8.4%.

**Fig. 1:**
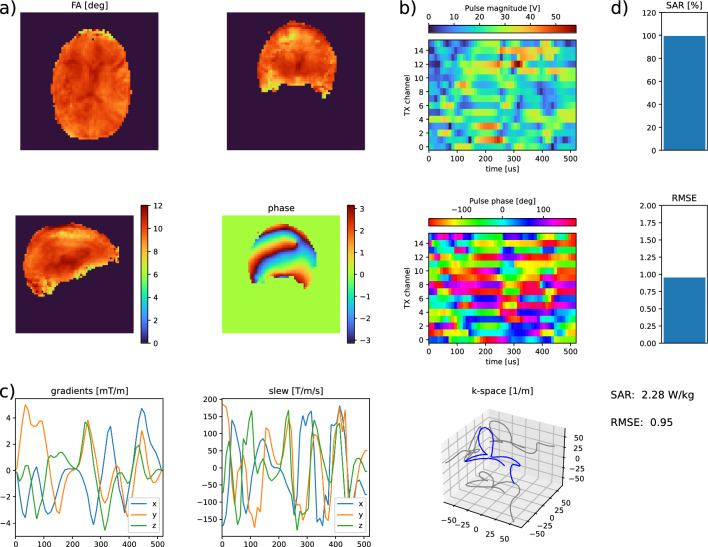
Optimization result of a non-selective 10° pTx pulse with a duration of 520 µs. The optimization took 66 s. Subplot (**a**) displays the simulated FA and the resulting phase. Subplot (**b**) shows the magnitude and phase of all RF waveforms. The gradient waveforms and the corresponding slew rates and k-space trajectories can be seen in subplot (**c**). If appropriate information such as sequence timing and target FA distribution is given, SAR and RMSE can be calculated and displayed (subplot (**d**))

**Fig. 2:**
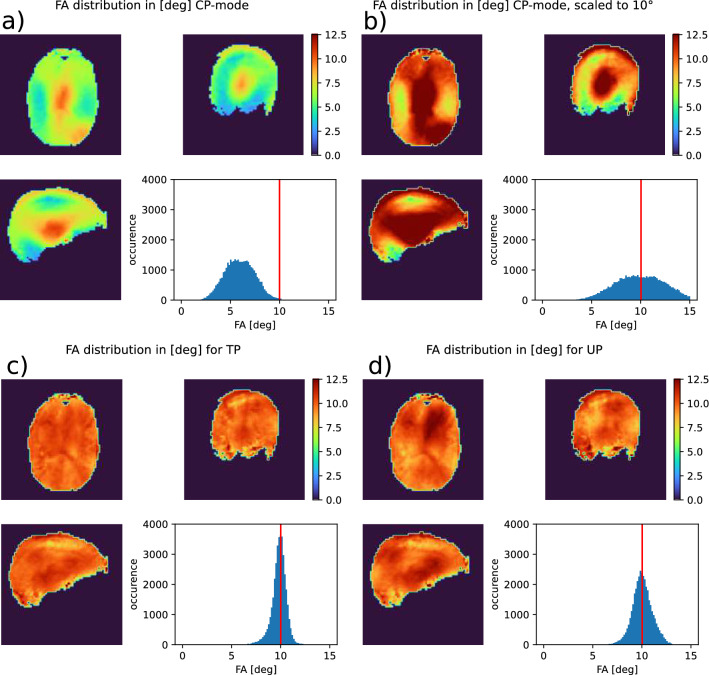
Simulated flip angle (FA) distribution in a subject that was not part of the UP pulse design database. **a**: CP mode rectangular pulse as calculated by the MR scanner. **b**: CP mode rectangular pulse scaled to a mean FA of 10°. **c**: Tailored non-selective pulse for this specific subject. **d**: Universal non-selective pulse. The CP mode pulse excites with a wide FA distribution. Additionally, the MR scanner scales RF power too low, so that the median FA is significantly lower than the target FA of 10°. Both pTx pulses excite with an appropriate median FA. As expected, the tailored pulse outperforms the universal pulse with regard to FA homogeneity

A full Bloch simulation, taking into account previously recorded $B_1^+$ and $B_0$ maps, was implemented. It allows for accurate simulation of arbitrary RF and gradient pulses. Additionally, to allow for expedited simulation of small tip angle pulses, a simulation based on the small tip angle approximation was also implemented. It follows the forward simulation of the *spatial domain method* [1], in which gradient waveforms, $B_0$ map and $B_1^+$ sensitivities are assembled into a system matrix (see Equation 3).

Using single-channel $B_1^+$ maps and a $B_0$ map of one or several subjects, the resulting FA distribution of a pTx RF pulse with underlying gradient pulse can be simulated using both methods. With consideration of the average repetition time of the RF pulses, their maximum local SAR can be calculated.

### 4. RF pulse optimization

All optimizations were run on a dedicated server for data reconstruction and pulse optimization which was equipped with a NVIDIA RTX A6000 GPU with 48 GiB of memory and a AMD Ryzen Threadripper PRO 3995WX 64-Core CPU. All optimizations were performed on the GPU unless stated otherwise. Optimization of RF and gradient waveforms was performed using the AdamW algorithm [22]. As it employs a gradient descent based method, the optimization greatly benefits from the autodifferentiation capabilities of PyTorch. The learning rate was set to $4\cdot 10^{-4}$ and the internal momentum parameters of the optimizer were reinitialized after every set of 1000 iterations, using the former best performing pulse as start value. Each sample and each channel of the RF and gradient waveforms was optimized freely, resulting in a very large number of parameters that needed to be optimized.

To respect hardware- and regulatory limits, a cost function was designed. It uses carefully chosen weights to balance different metrics, which were determined experimentally.

4 $$\begin{aligned} \begin{aligned} c(p,g) =\,&pen_\text {rmse}(g,p) + err_\text {volt}(p) + err_\text {maxG}(g) + err_\text {slew}(g) + err_\text {edge}(p,g) \\ +&pen_\text {power}(p) + pen_\text {slew}(g) + err_\text {sar}(p) \end{aligned} \end{aligned}$$

All elements of the cost function depend on the gradients *g*, the RF-pulse *p* or both. It consists of penalty terms (named $pen_{\ldots }$), which have a relatively low magnitude that is usually positive non-zero and scales with the input, and of error terms (named $err_{\ldots }$) which have a value of zero until a certain limit is exceeded. The exact equations for all terms are listed in Table 1. By defining a target flip angle distribution, a pulse duration, and a sampling interval, pTx pulses can be optimized using either the small tip angle approximation or the Bloch simulation.

Based on the $B_1^+$ and $B_0$ maps of the first 9 subjects, a universal pulse (UP) with a nominal flip angle of 10° and a SAR limit of 2.3 W/kg at a TR of 19.7 ms was designed. Pulse duration was 520 µs with a raster time of 10 µs. Optimization of all three gradient axes and 16 complex RF waveforms (in total 1820 real-valued parameters) was run on the small tip angle model for 7,500 iterations. Subsequently, the RF waveforms were refined by 2,500 steps of optimization based on the Bloch model, using the RF and gradient waveforms of the STA optimization as a starting point. By designing the pulse for 10°  it was possible to scale it down to the required flip angle without risk of exceeding SAR or power limits. Following the small tip angle approximation, the pulse flip angle could be scaled linearly by scaling the pulse voltage. This allowed a single universal pulse to be designed and used for different applications requiring different small flip angles, making the pulse even more universal.

**Fig. 3:**
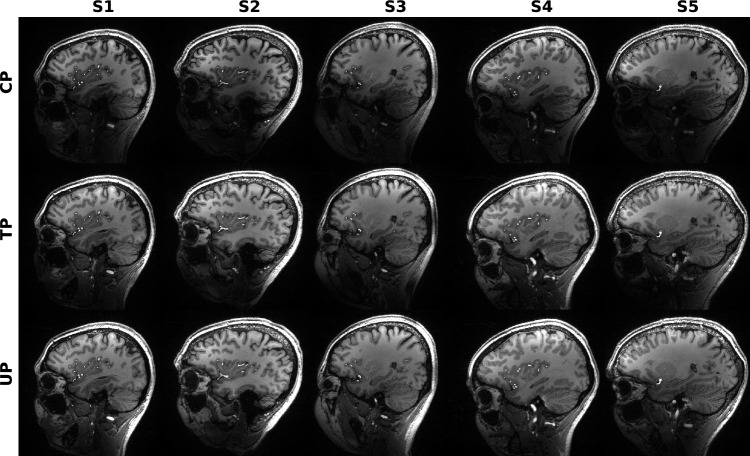
Sagittal view of MPRAGE brain images of 5 different subjects. The first row (“CP”) shows images using a rectangular CP mode pulse for excitation. In the second row (“TP”) a non-selective subject-specific tailored excitation pulse was used. In the third row (“UP”) excitation was done with a non-selective universal pulse which was designed on 9 subjects. CP mode excitation creates severe image inhomogeneities, e.g., near the cerebellum. This is greatly reduced in the images acquired with either of the pTx pulses. None of the subjects displayed here was part of the UP design database

**Fig. 4:**
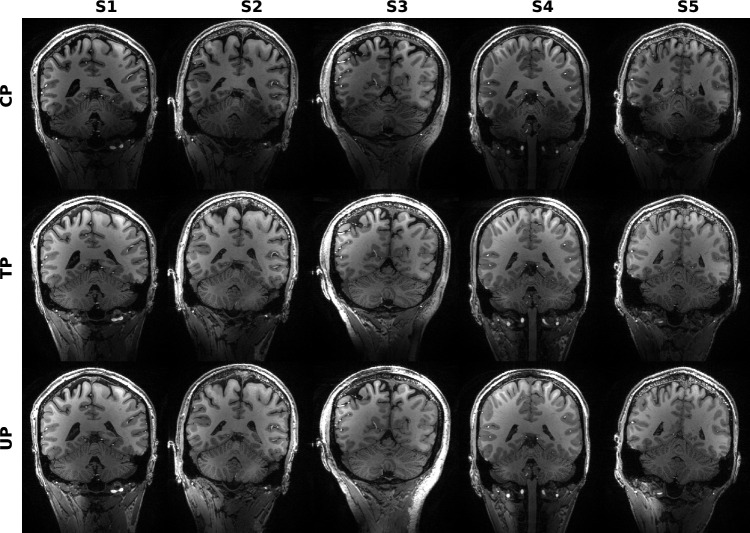
Coronal view of MPRAGE brain images of 5 different subjects. The first row (“CP”) shows images using a rectangular CP mode pulse for excitation. In the second row (“TP”) a non-selective subject-specific tailored excitation pulse was used. In the third row (“UP”) excitation was done with a non-selective universal pulse which was designed on 9 subjects. CP mode excitation creates severe image inhomogeneities, e.g., near the cerebellum. This is greatly reduced in the images acquired with either of the pTx pulses. None of the subjects displayed here was part of the UP design database

**Fig. 5:**
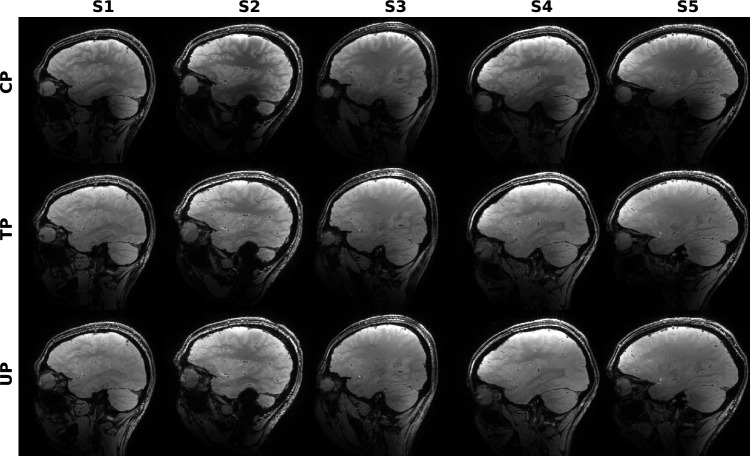
Saggital view of GRE brain images of 5 different subjects. The same excitation pulses as for the MPRAGE sequence (Figs. 2 and 3) were used, scaled to 5° flip angle. Similar inhomogeneities near the cerebellum can be observed for the CP mode pulses, while both pTx pulses improved homogeneity

For five additional subjects that were not part of the UP design database, a TP was designed using the same design criteria. In this case, however, optimization was performed on the small tip angle model only to keep computation time short and stopped after 2,500 iterations. For both pulses, Bloch simulation was used to evaluate the pulses’ performance.

Furthermore, to test the toolbox’s performance in the high-FA-regime, a 90° pulse was designed. This was done by first designing a 9° pulse using the STA, under appropriately scaled peak power constraints, for 3500 iterations. The RF waveforms were then scaled up by a factor of 10 and refined with 1000 iterations of the Bloch simulation, optimizing the RF waveforms only. This pulse was designed without SAR constraint since it was only played out once, as preparation pulse for a satTFL sequence.

As a benchmark for the free waveform tailored pulse calculated with FastPtx, a 3 kT point pulse was generated for a single subject. The optimization was performed similarly to the spatial domain method [1] using the variable exchange method [3] to solve the magnitude-least-squares problem with 20 iterations on an unregularized minimization. The k-space location of the first two kT points was chosen by randomly generating 5,000 pairs of k-space positions in the range $-14\,m^{-1} \le k_{x,y,z} \le 14\,m^{-1}$ and selecting the one that gave the best performance for the given scenario. The third kT point was positioned in the center of the k-space. The three sub pulses were designed to be 130 µs long with a pause of 60 µs between sub pulses for gradient blips.

### 5. MPRAGE measurements

A 3D MPRAGE protocol was set up with the following parameters: TR1=3,360 ms, TR2=6.16 ms, TE=3.03 ms, BW=420 Hz/px, matrix size=264x264x224, resolution=0.8 mm isotropic, GRAPPA 2x2, Inversion Time=1,340 ms, FA=9°. The voltage of the adiabatic inversion pulse (HS4, 12.8ms) was scaled to 460V, so that it attributed to $\approx$7 W/kg of local SAR.

For excitation, three different pulses were used. The first protocol (MPRAGE_CP_) used a conventional rectangular pulse in CP mode. For the second protocol (MPRAGE_UP_), the universal pulse was scaled down to 9° and used as excitation pulse. The third protocol (MPRAGE_TP_) used the subject-specific, tailored pTx pulse which was also scaled down to 9°. All three protocols used the adiabatic HS4 pulse for inversion.

All three excitation pulses were applied on 5 subjects which were not in the design database of the universal pulse and results were compared.

### 6. GRE measurements

To obtain images with a T2* weighted contrast, 3D GRE images with the following parameters were acquired: TR=11 ms, TE=7 ms, BW=260 Hz/px, matrix size=264x264x224, resolution=0.8 mm isotropic, GRAPPA 2x2, FA=5°.

The excitation pulse was varied similarly as for the MPRAGE sequence: The first protocol (GRE_CP_) used a conventional rectangular pulse, second protocol (GRE_UP_) the universal pulse scaled down to 5°, and the third protocol (GRE_TP_) used the subject-specific, tailored pTx pulse which was also scaled down to 5°. All three pulses were applied on the same 5 subjects which were not in the design database of the universal pulse.

### 7. Simulation experiment

In a simulation-only experiment on a single-subject dataset, the CP mode pulse used for the MPRAGE sequence was scaled to a mean FA of 10°, which resulted in 0.92 W/kg of local SAR. Using this as a SAR constraint for optimization, a pTx pulse was designed with the goal of outperforming the CP mode pulse while maintaining the same SAR budget. Optimization parameters were comparable to those for the tailored pulses on single subjects.

### 8. Slab selective excitation

A slab-selective pulse (thickness 50 mm, orientation transversal$\rightarrow$ coronal 45°, FA 10°) was designed starting from a conventional CP mode sinc pulse with a trapezoidal slice-selection gradient. Parameters were: time-bandwidth product 8, pulse duration 1.2 ms, total duration (including gradients) 2.4 ms. The pulse was used as a starting point for free optimization of ptx RF and gradient waveforms with the goal of FA homogenization. A universal pTx pulse was designed using the same design database as before. Optimization was run for 20,000 iterations.

**Fig. 6:**
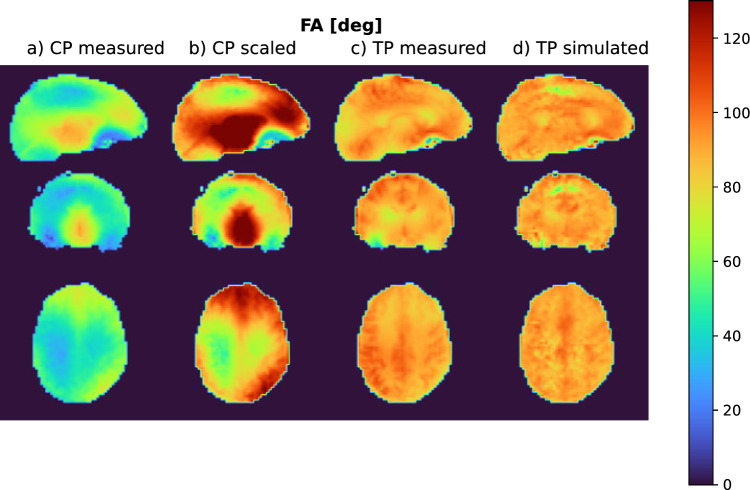
A 90° rectangular CP mode pulse scaled by the MRI system (**a**) and scaled up to an average FA of 90°(**b**), compared to a 90° tailored pTx pulse (TP, **c**), both measured using a satTFL sequence. The right column (**c**) shows the simulated FA distribution of the 90° TP. Anatomic features are visible in all three maps, which is likely to be an artifact from the utilized $B_1^+$ mapping sequence

**Fig. 7:**
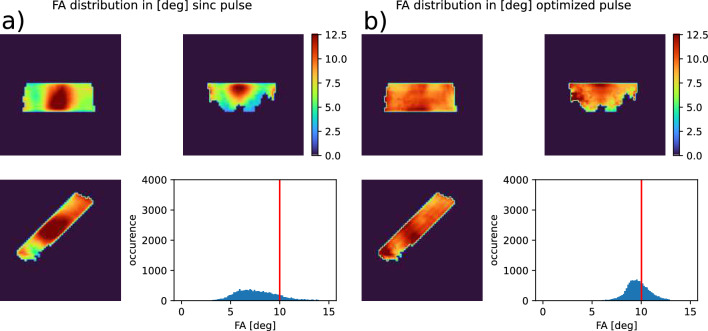
Simulated flip angle (FA) distribution of a slab selective pulse in a subject that was not part of the UP pulse design database. **a**: CP mode sinc pulse as calculated by the MR scanner. **b**: Universal pTx pulse. The CP mode pulse excites with a wide FA distribution. Additionally, the MR scanner scales RF power too low, so that the median FA is significantly lower than the target FA of 10°. The universal pulse excites more homogeneously with the mean FA closer to the target than in the sinc pulse

**Fig. 8:**
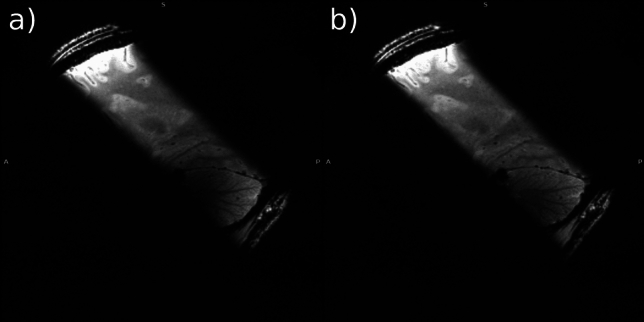
GRE images recorded with slab selective pulses. **a**: CP mode sinc pulse. **b**: optimized pTx UP. While both pulses achieve a clean slice profile, excitation in the cerebellum is more homogeneous with the pTx pulse

The so designed pulse was then applied on a subject which was not part of the design database. The same 3D GRE sequence was used as for non-selective excitation. This allowed not only to observe the excited area, but also to get an impression of the slice profile since the entire brain was encoded in the resulting image. FA distribution and image were compared to the sinc pulse.

### 9. Robustness of pulses

A potential source of error in pTx excitation is a lack of stability in the transmit path of the MR scanner. To investigate the effect of slightly imprecise Tx channels, a simulation experiment was performed. A magnitude factor and a phase offset were generated for each channel. These parameters were drawn from a Gaussian normal distribution with a standard deviation of 5% for the amplitude and 5° for the phase. These values were chosen because they are similar to the accuracies the scanner manufacturer specifies for SAR supervision measurements (12% power, 5° phase on 7 tesla systems), implying that these are the maximum deviations to be expected when playing out RF pulses. The UP and CP mode pulse were then adjusted by these values and simulated on $B_1^+$ maps from a subject who was not part of the UP design database. To get a wide range of magnitude and phase error combinations, this experiment was repeated 10 times.

While this issue is not related to our toolbox but instead affects all applications, it is important to get an understanding of the scale of the effects caused by those imprecisions. In contrast to the extensive experience with conventional pulse shapes, there is little experience with freely designed RF waveforms and their stability, so this simulation experiment can give an estimate of the impact of instabilities on the pulses generated by FastPtx.

## Results

### 1. pTx pulse design

Calculation of the 10° UP took 1225 s for 9 subjects. The resulting pulse shapes, k-space trajectory, and simulated excitation pattern are displayed in Fig.  1. When simulating the resulting pulse, the NRMSE between simulation and target FA was 11.2% for both the STA simulation and the Bloch simulation. The resulting FA distributions of STA and Bloch simulation differed with a NRMSE of 0.62%, making them virtually identical. When simulating the UP on the 5 subjects which were not part of the UP design database, the NRMSE to the target FA was (12.3±1.7)% (mean±standard deviation across all subjects’ NRMSEs).

The tailored pulses for the same 5 subjects took approx. 67 s each to optimize and resulted in a NRMSE of (8.2±1.0)%. In comparison, the CP mode pulses which excite with an NMRSE of (42.0±1.4)%. Figure 2 compares the flip angle distribution for all three pulses in the same subject. It can be clearly seen that the CP mode pulse not only has the widest distribution, but also misses the mean target FA of 10° by circa 40%. As expected, the tailored pulse performs best, with a very narrow FA distribution.

When disabling the GPU for tailored pulse optimization, the optimization time increased to 884 s, showing the importance of GPU computing in multi-parameter optimization problems.

The 3 kT point pulse had a total duration of 510 µs and resulted in an excitation with a nRMSE of 15.1%, while the FastPtx TP on the same subject resulted in an nRMSE of 6.8%.

### 2. Imaging sequences

Figures 3 and 4 depict sagittal and coronal slices, respectively, for each subject, which were acquired using the MPRAGE sequence. In these slices, there are regions where the CP mode pulses excited insufficiently, while both pTx pulses were effective. These differences in excitation were particularly noticeable in the cerebella.

The same effect also becomes visible in the GRE images (Fig. 5). Here the gray-white-matter contrast in the CP mode images is stronger than in the pTx images. An experiment performed later showed that the contrast could be restored by reducing the FA of the pTx images to 3°, which is the actual mean FA of the CP mode acquisitions.

### 3. Large-FA pulse

The 90° pulse was used as preparation pulse in a satTFL sequence. Optimization took 336 sec. In simulation it achieved an NRMSE of 7.1%. The measured FA map is shown in Fig. 6. We measured an NRMSE of 8.35%, compared to the CP mode’s NRMSE of 39.58%. The mean FA was measured at 88.6° (TP) compared to 52.2° (CP).

### 4. Simulation-only pulse design

When we scaled up the CP mode pulse to 10° FA, we observed a peak local SAR of 0.92 W/kg. Using this as a SAR constraint for a pTx pulse of the same length, we were able to reduce the NRMSE from 23.9% with the scaled CP mode pulse to 12.2% with the pTx pulse.

### 5. Slab selective excitation

Design of the slab selective pTx pulse took 1 h and 25 min. In simulations, the slab-selective CP mode sinc pulse achieved an RMSE of 3.20° at 10° nominal FA. The freely designed pTx pulse achieved an RMSE of 1.67°. FA maps of both pulses are displayed in Fig. 7. Figure 8 shows in-vivo images using the pulses.

### 6. Robustness of pulses

Adding random phase and magnitude offsets to each channel did influence the resulting NRMSE. The original UP achieved a NRMSE of 13.08% in this subject. With magnitude and phase errors added, NMRSEs between 12.44% and 14.79% were achieved. The CP mode pulses, in comparison originally achieved a NRMSE value of 39.89%. With errors added, RMSEs between 39.67% and 41.66% were achieved.

## Discussion

We developed a toolbox that uses the PyTorch framework for the optimization of RF and gradient waveforms. Since Pytorch has autodifferentiation capabilities and allows to easily utilize a powerful GPU for computation and optimization, the required time for pTx pulse optimization could be reduced drastically, therefore allowing to optimize three gradient waveforms and 16 complex RF waveforms on-line during an experiment. The toolbox was used to optimize subject-specific tailored pulses (TPs), which have been shown to be more effective than universal pulses (UPs) that are optimized using a representative subject cohort. We demonstrate the use of the toolbox by acquiring MPRAGE and GRE images using both a UP and a TP that was optimized in about one minute.

While UP generation was possible and delivered good results, TPs could be generated within short time, increasing FA homogeneity even further. The fast calculation of pulses allows for optimization of exotic excitation patterns, where UPs do not exist or are too general. Examples of possible applications include TONE-pulses (previously implemented as pTx 2-spoke pulses [23]) and inner-volume-excitation pulses (commonly implemented using predefined gradient trajectories, resulting in long pulses).

The CP mode pulses produced by the scanner’s software were found to have a FA that was 40% lower than expected. To improve the FA distribution around the target FA, one simple solution would be to increase the pulse power. While this method may not produce an FA distribution as good as that of pTx pulses, it may be sufficient for some applications and eliminate the need for more complex solutions. This also has the consequence that protocols which were optimized on the scanner’s CP mode pulses might have to be adjusted when utilizing pTx pulses to adjust for the 40% difference in flip angle.

The 3 kT points pulse we generated caused a nRMSE that was approximately twice as high as the one from the FastPtx pulse. As there were no SAR constraints implemented in the kT point optimization, we expect this to get even worse when taking SAR into account.

We successfully demonstrated that our optimization toolbox can handle large FA pulses. By utilizing the approach of optimizing a small-FA pulse with the STA, scaling it up, and then refining it with Bloch simulations, we were able to obtain a useful 90° pulse.

During pulse design SAR is calculated with the same VOP file the scanner uses for online SAR supervision. This allows for an accurate SAR prediction and optimal usage of the allowed energy disposition into the tissue.

In simulation, we demonstrated the effect of small deviations of the transmitters from their nominal behavior. While a phase and amplitude deviation in the order of 5% and 5° respectively degraded the performance of the universal pulse, this was also the case for the commonly used CP mode pulse. The pTx pulse still showed good performance, demonstrating that it is not overly sensitive to small hardware imperfections. These simulations, however, assumed constant magnitude and phase offsets for the entire duration of the RF pulse. It is, however, possible that RF phase or magnitude drift on a much shorter timescale while the RF pulse is being played out. Effects of such instabilities should be investigated further.

Experiments in different scenarios such as additional $B_0$ field strengths or with other target excitation profiles could help to find the limitations of the toolbox provided. In case of the tailored pTx pulses for non-selective excitation, $B_1^+$ mapping took significantly longer compared to the pulse design duration. This time could be reduced by other techniques such as machine-learning supported $B_1^+$ mapping [24].

While Luo et al [7] have already published a toolbox using autodifferentiation for simultaneous RF- and gradient waveform optimization, our implementation adds the ability to optimize parallel transmit pulses. Our implementation also had to take SAR prediction based on VOP files into account, to generate only pTx pulses that could also be played out on the scanner. The implementation by Luo et al also relies on analytic expressions for Jacobians in some places, which makes the computation more efficient, but also does not allow to easily change the code of these sections without re-determining the Jacobians. The lack of analytic Jacobians makes our code run less efficiently, but significantly more flexible. We were able to integrate a small tip angle solver which has proven useful with comparably little effort.

Future improvements of this toolbox could be achieved by providing explicit Jacobian operations for the Bloch simulations, as was done by Luo et al. This could potentially be done with relatively low effort since Luo et al published their source code. Since the two packages were developed independently it would still require some integration work. This could potentially speed up optimization and improve convergence of the optimized RF- and gradient pulses. Additionally, usability could be improved by fully integrating the toolbox into the scanner’s environment, thus eliminating manual steps for pTx calibration.

## Data Availability

The source code of the FreePtx python toolbox as well as the $B_1^+$/$B_0$ maps required to reproduce this work are available under https://github.com/dabosch/FastPtx.

## References

[1] Grissom W, Yip Cy, Zhang Z, Stenger VA, Fessler JA, Noll DC (2006). Spatial domain method for the design of RF pulses in multicoil parallel excitation. Magn Reson Med.

[2] Vinding MS, Guérin B, Vosegaard T, Nielsen NC (2017). Local SAR, global SAR, and power-constrained large-flip-angle pulses with optimal control and virtual observation points. Magn Reson Med.

[3] Setsompop K, Wald LL, Alagappan V, Gagoski BA, Adalsteinsson E (2008). Magnitude least squares optimization for parallel radio frequency excitation design demonstrated at 7 Tesla with eight channels. Magn Reson Med.

[4] Geldschläger O, Bosch D, Henning A (2022). OTUP workflow: target specific optimization of the transmit k-space trajectory for flexible universal parallel transmit RF pulse design. NMR Biomed.

[5] Herrler J, Liebig P, Gumbrecht R, Ritter D, Schmitter S, Maier A, Schmidt M, Uder M, Doerfler A, Nagel AM (2021). Fast online-customized (FOCUS) parallel transmission pulses: a combination of universal pulses and individual optimization. Magn Reson Med.

[6] Hao S, Fessler JA, Noll DC, Nielsen JF (2016). Joint design of excitation k-space trajectory and RF pulse for small-tip 3D tailored excitation in MRI. IEEE Trans Med Imaging.

[7] Luo T, Noll DC, Fessler JA, Nielsen JF (2021). Joint design of RF and gradient waveforms via auto-differentiation for 3D tailored excitation in MRI. IEEE Trans Med Imaging.

[8] Gras V, Vignaud A, Amadon A, Bihan DL, Boulant N (2016). Universal pulses: a new concept for calibration-free parallel transmission. Magn Reson Med.

[9] Geldschläger O, Bosch D, Glaser S, Henning A (2021). Local excitation universal parallel transmit pulses at 9.4T. Magn Reson Med.

[10] Majewski K (2021). Simultaneous optimization of radio frequency and gradient waveforms with exact Hessians and slew rate constraints applied to kT-points excitation. J Magn Reson.

[11] Paszke A, Gross S, Chintala S, Chanan G, Yang E, DeVito Z, Lin Z, Desmaison A, Antiga L, Lerer A (2017) Automatic differentiation in PyTorch. 31st conference on neural information processing systems

[12] Paszke A, Gross S, Massa F, Lerer A, Bradbury J, Chanan G, Killeen T, Lin Z, Gimelshein N, Antiga L, Desmaison A, Köpf A, Yang E, DeVito Z, Raison M, Tejani A, Chilamkurthy S, Steiner B, Fang L, Bai J, Chintala S (2019). PyTorch: an imperative style, high-performance deep learning. Library.

[13] Pauly J, Nishimura D, Macovski A (1989). A k-space analysis of small-tip-angle excitation. J Magn Reson (1969).

[14] Shajan G, Kozlov M, Hoffmann J, Turner R, Scheffler K, Pohmann R (2013). A 16-channel dual-row transmit array in combination with a 31-element receive array for human brain imaging at 9.4 T. Magn Reson Med.

[15] Bosch D, Bause J, Ehses P, Zaiss M, Scheffler K (2020) In: Proc. Intl. Soc. Mag. Reson. Med. 28 (Virtual, 2020), p. 3703

[16] Bosch D, Bause J, Geldschläger O, Scheffler K (2023). Optimized ultrahigh field parallel transmission workflow using rapid presaturated TurboFLASH transmit field mapping with a three-dimensional centric single-shot readout. Magn Reson Med.

[17] Brunner DO, Pruessmann KP (2009). B1+ interferometry for the calibration of RF transmitter arrays. Magn Reson Med.

[18] Griswold MA, Jakob PM, Heidemann RM, Nittka M, Jellus V, Wang J, Kiefer B, Haase A (2002). Generalized autocalibrating partially parallel acquisitions (GRAPPA). Magn Reson Med.

[19] Eichfelder G, Gebhardt M (2011). Local specific absorption rate control for parallel transmission by virtual observation points. Magn Reson Med.

[20] Christ A, Kainz W, Hahn EG, Honegger K, Zefferer M, Neufeld E, Rascher W, Janka R, Bautz W, Chen J, Kiefer B, Schmitt P, Hollenbach HP, Shen J, Oberle M, Szczerba D, Kam A, Guag JW, Kuster N (2009). The virtual family—development of surface-based anatomical models of two adults and two children for dosimetric simulations. Phys Med Biol.

[21] Orzada S, Fiedler TM, Quick HH, Ladd ME (2021). Local SAR compression algorithm with improved compression, speed, and flexibility. Magn Reson Med.

[22] Loshchilov I, Hutter F (2019). Decoupled weight decay regularization. 10.48550/arXiv.1711.05101

[23] Saib G, Gras V, Mauconduit F, Boulant N, Vignaud A, Brugières P, Le Bihan D, Le Brusquet L, Amadon A (2019). Time-of-flight angiography at 7T using TONE double spokes with parallel transmission. Magn Reson Imaging.

[24] Eberhardt B, Poser BA, Shah NJ, Felder J (2022). B1 field map synthesis with generative deep learning used in the design of parallel-transmit RF pulses for ultra-high field MRI. Z Med Phys.

